# Supplementary material for: Population structure-guided profiling of antibiotic resistance patterns in clinical Listeria monocytogenes isolates from Germany identifies pbpB3 alleles associated with low levels of cephalosporin resistance
Source: Emerg Microbes Infect. 2020 Aug 5;9(1):1804–13. doi: 10.1080/22221751.2020.1799722 (PMC7473133; doi:10.1080/22221751.2020.1799722)
Supplement: Fischer_et_al._supplementary_material_finanl.doc [file TEMI_A_1799722_SM4653.doc]

number

| 250 |  |  |  |  |  |  |  |  |  |  |  |  |
| --- | --- | --- | --- | --- | --- | --- | --- | --- | --- | --- | --- | --- |
|  |  |  |  |  |  |  |  |  |  |  |  |  |
| 200 |  |  |  |  |  |  |  |  |  |  |  | IIa |
|  |  |  |  |  |  |  |  |  |  |  |  |  |
|  |  |  |  |  |  |  |  |  |  |  |  |  |
|  |  |  |  |  |  |  |  |  |  |  |  |  |
|  |  |  |  |  |  |  |  |  |  |  |  |  |
|  |  |  |  |  |  |  |  |  |  |  |  |  |
| 150 |  |  |  |  |  |  |  |  |  |  |  | IIb |
|  |  |  |  |  |  |  |  |  |  |  |  |  |
|  |  |  |  |  |  |  |  |  |  |  |  |  |
|  |  |  |  |  |  |  |  |  |  |  |  | IIc |
|  |  |  |  |  |  |  |  |  |  |  |  |  |
|  |  |  |  |  |  |  |  |  |  |  |  |  |
|  |  |  |  |  |  |  |  |  |  |  |  |  |
| 100 |  |  |  |  |  |  |  |  |  |  |  | IVa |
|  |  |  |  |  |  |  |  |  |  |  |  |  |
|  |  |  |  |  |  |  |  |  |  |  |  | IVb |
|  |  |  |  |  |  |  |  |  |  |  |  | IVb-v1 |
|  |  |  |  |  |  |  |  |  |  |  |  |  |
|  |  |  |  |  |  |  |  |  |  |  |  |  |
|  |  |  |  |  |  |  |  |  |  |  |  |  |
| 50 |  |  |  |  |  |  |  |  |  |  |  | IVc |
|  |  |  |  |  |  |  |  |  |  |  |  |  |
|  |  |  |  |  |  |  |  |  |  |  |  |  |
|  |  |  |  |  |  |  |  |  |  |  |  |  |
|  |  |  |  |  |  |  |  |  |  |  |  |  |
|  |  |  |  |  |  |  |  |  |  |  |  |  |

0

2009 2010 2011 2013 2014 2015 2016 2017 2018 2019

isolation year

**Figure S1:** Isolation year in the selection of 544 clinical *L. monocytogenes* isolates that were subjected to antibiotic susceptibility testing.


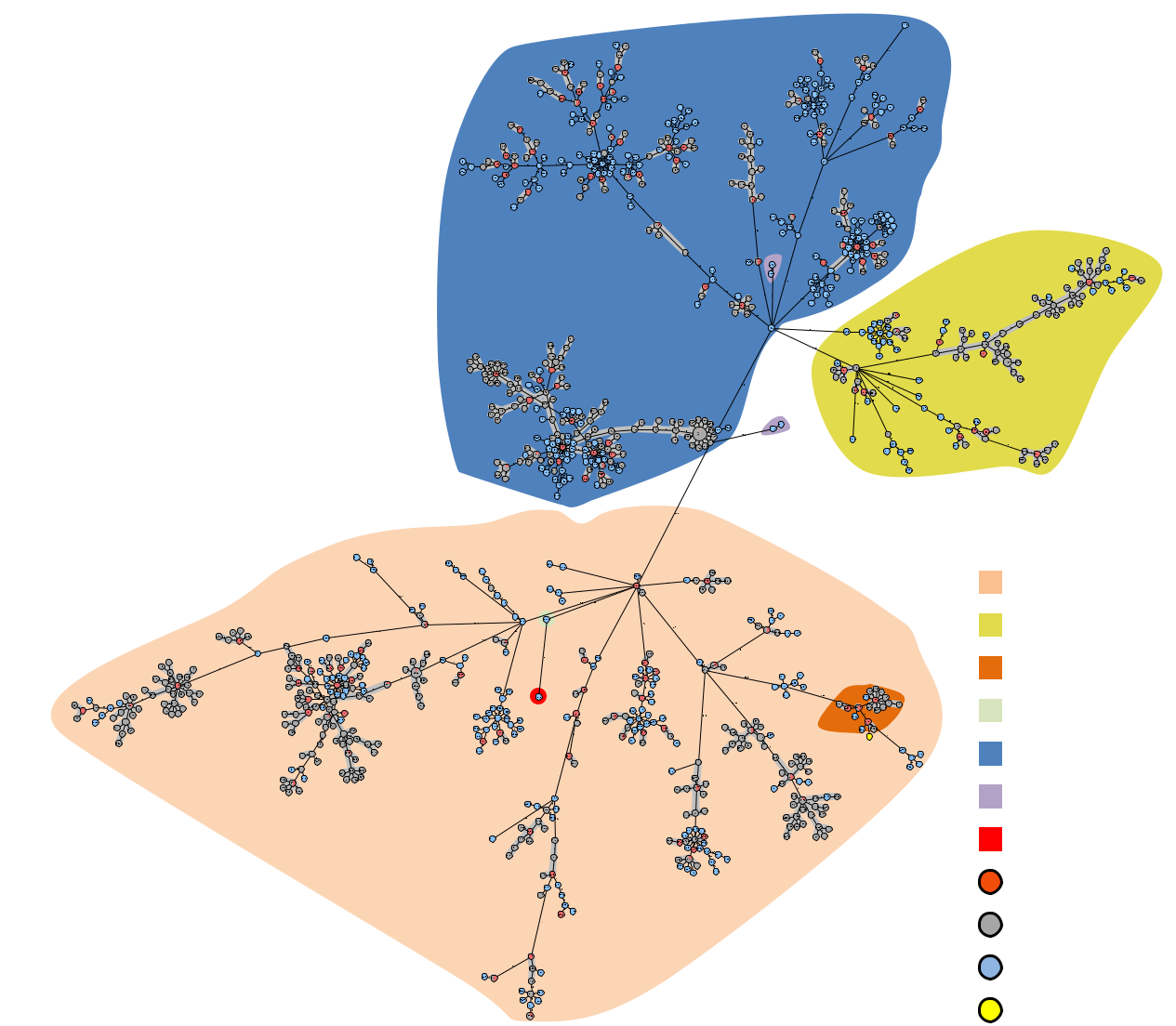


IIa

IIb

IIc

IVa

IVb

IVb-1

IVc

cluster representatives

cluster associated

singletons

EGD-e

**Figure S2:** Minimum spanning tree of 1220 clinical *L. monocytogenes* isolates based on their cgMLST profiles. Samples screened for antimicrobial resistance patterns are colorized in red (outbreak cluster representatives) and blue (single isolates). The background is colorized according to molecular PCR serogroups.

|  | 100 |  |  |  |  |  |  |  |  |  |  |  |  |  |  |  |  |  |
| --- | --- | --- | --- | --- | --- | --- | --- | --- | --- | --- | --- | --- | --- | --- | --- | --- | --- | --- |
|  | 90 |  |  |  |  |  |  |  |  |  |  |  |  |  |  |  |  |  |
|  | 80 |  |  |  |  |  |  |  |  |  |  |  |  |  |  |  |  |  |
| number | 70 |  |  |  |  |  |  |  |  |  |  |  |  |  |  |  |  |  |
|  | 60 |  |  |  |  |  |  |  |  |  |  |  |  |  |  |  |  |  |
|  | 50 |  |  |  |  |  |  |  |  |  |  |  |  |  |  |  |  |  |
|  | 40 |  |  |  |  |  |  |  |  |  |  |  |  |  |  |  |  |  |
|  | 30 |  |  |  |  |  |  |  |  |  |  |  |  |  |  |  |  |  |
|  | 20 |  |  |  |  |  |  |  |  |  |  |  |  |  |  |  |  |  |
|  | 10 |  |  |  |  |  |  |  |  |  |  |  |  |  |  |  |  |  |
|  | 0 |  |  | 37 |  |  | 87 |  |  | 182954 |  | 591421 |  | 1691 |  |  |  | 203226 |
|  | 1628 | 451 | 4 |  | 735 | N.A.155 |  | 403399121 | 9 |  | 219217 |  | 224204101388412177 |  | 249 | 382 | 504431200 |  |

MLST ST

**Figure S3:** MLST sequence types (STs) in the selection of 544 clinical *L. monocytogenes* isolates that were subjected to antibiotic susceptibility testing. Only STs with more than one strain are shown.

| ampicillin |  | penicillin |
| --- | --- | --- |
| 140 | 160 |  |
| 120 | 140 |  |
| 100 | 120 | IIa |
|  | 100 |  |
| 80 |  |  |
|  |  |  |
|  | 80 | IIb |
| 60 |  |  |
|  | 60 | IVb |
| 40 |  |  |
|  | 40 |  |
|  |  |  |
|  |  |  |
| 20 | 20 |  |
| 0 | 0 |  |


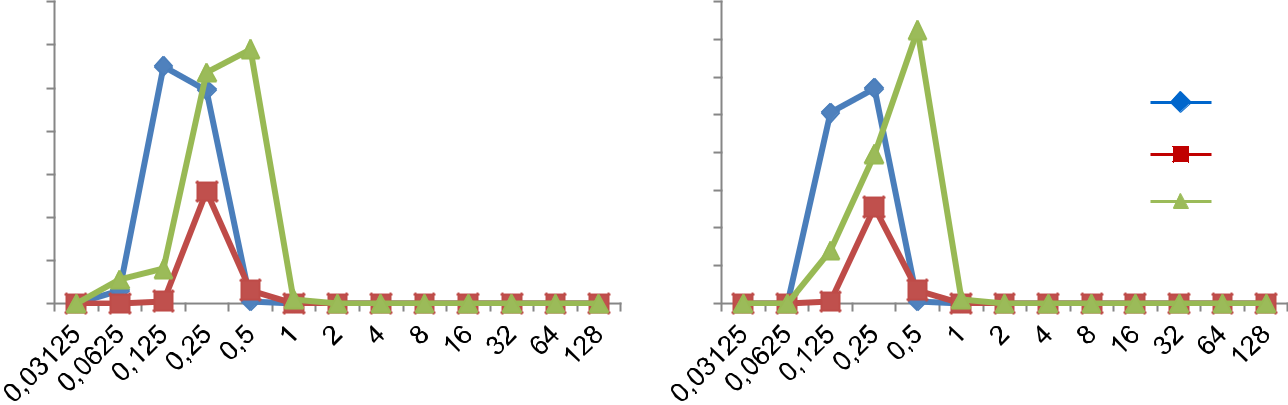


| daptomycin |  | linezolid |
| --- | --- | --- |
| 160 | 250 |  |
| 140 | 200 |  |
| 120 |  |  |
|  |  | IIa |
| 100 | 150 |  |
|  |  |  |
|  |  |  |
| 80 | 100 | IIb |
| 60 |  | IVb |
|  |  |  |
| 40 |  |  |
|  | 50 |  |
| 20 |  |  |
|  |  |  |
| 0 | 0 |  |


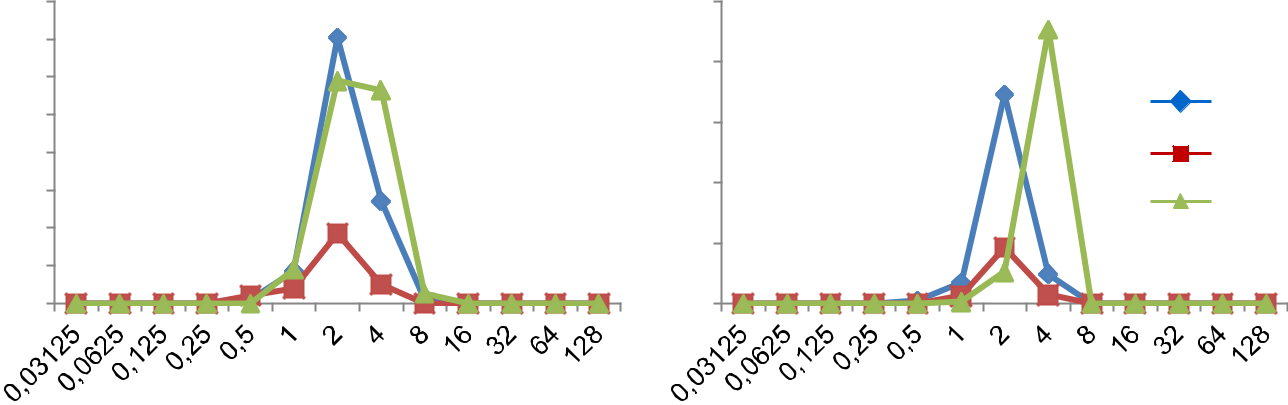


| tetracycline |  | tigecycline |
| --- | --- | --- |
| 200 | 250 |  |
| 180 |  |  |
| 160 | 200 |  |
| 140 |  | IIa |
| 120 | 150 |
|  |
| 100 |  | IIb |
| 80 | 100 | IVb |
| 60 |  |
|  |  |
| 40 | 50 |  |
| 20 |  |  |
| 0 | 0 |  |
| MIC [mg/l] |  | MIC [mg/l] |


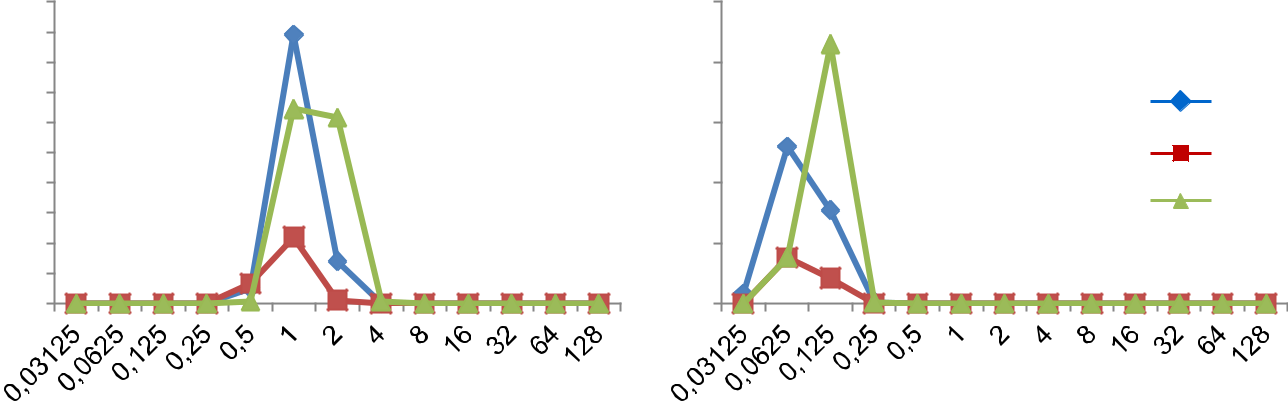


**Figure S4:** Distribution of minimal inhibitory concentrations for different antibiotics in *L.* *monocytogenes* isolates belonging to molecular PCR serogroups IIa (n=216), IIb (n=59) and IVb (n=254).
